# Circulation of a Quorum-Sensing-Impaired Variant of *Vibrio cholerae* Strain C6706 Masks Important Phenotypes

**DOI:** 10.1128/mSphere.00098-16

**Published:** 2016-05-25

**Authors:** Sandrine Stutzmann, Melanie Blokesch

**Affiliations:** Laboratory of Molecular Microbiology, Global Health Institute, School of Life Sciences, École Polytechnique Fédérale de Lausanne (EPFL), Lausanne, Switzerland; University of Kentucky

**Keywords:** *Vibrio cholerae*, *luxO* mutation, natural competence for transformation, quorum sensing, type VI secretion system

## Abstract

*Vibrio cholerae*, the causative agent of cholera, is a model organism for studying virulence regulation, biofilm formation, horizontal gene transfer, and the cell-to-cell communication known as quorum sensing (QS). As in any research field, discrepancies between data from diverse laboratories are sometimes observed for *V. cholerae*. Such discrepancies are often caused by the use of diverse patient or environmental isolates. In this study, we investigated the inability of a few laboratories to reproduce high levels of natural transformation, a mode of horizontal gene transfer that is specifically induced on chitinous surfaces. This irreproducibility was mostly related to one specific isolate of *V. cholerae*: the O1 El Tor C6706 strain. C6706 was previously described as QS proficient, an important prerequisite for the induction of natural competence for transformation. To elucidate the underlying problem, we collected seven isolates of the same C6706 strain from different research laboratories in North America and Europe and compared their phenotypes. Importantly, we observed a split response with respect to QS-related gene expression, including chitin-induced natural competence and type VI secretion (T6S). While approximately half of the strains behaved as reported for several other O1 El Tor pandemic isolates that are commonly studied in the laboratory, the other half were significantly impaired in QS-related expression patterns. This impairment was caused by a mutation in a QS-related gene (*luxO*). We conclude that the circulation of such QS-impaired wild-type strains is responsible for masking several important phenotypes of *V. cholerae*, including natural competence for transformation and T6S.

**IMPORTANCE** Phenotypic diversity between laboratory-domesticated bacterial strains is a common problem and often results in the failed reproduction of published data. However, researchers rarely compare such strains to elucidate the underlying mutation(s). In this study, we tested one of the best-studied *V. cholerae* isolates, O1 El Tor strain C6706 (a patient isolate from Peru), with respect to two main phenotypes: natural competence for transformation and type VI secretion. We recently demonstrated that the two phenotypes are coregulated and specifically induced upon the growth of pandemic *V. cholerae* O1 El Tor strains on chitinous surfaces. We provide evidence that of seven C6706 strains collected from different laboratories, four were impaired in the tested phenotypes due to a mutation in a QS gene. Collectively, our data indicate that the circulation of such a mutated wild-type strain of C6706 might have had important consequences for QS-related data.

## Abstract (precis)

Phenotypic diversity between laboratory-domesticated bacterial strains is a common problem and often results in the failed reproduction of published data. However, researchers rarely compare such strains to elucidate the underlying mutation(s). In this study, we tested one of the best-studied *V. cholerae* isolates, O1 El Tor strain C6706 (a patient isolate from Peru), with respect to two main phenotypes: natural competence for transformation and type VI secretion. We recently demonstrated that the two phenotypes are coregulated and specifically induced upon the growth of pandemic *V. cholerae* O1 El Tor strains on chitinous surfaces. We provide evidence that of seven C6706 strains collected from different laboratories, four were impaired in the tested phenotypes due to a mutation in a QS gene. Collectively, our data indicate that the circulation of such a mutated wild-type strain of C6706 might have had important consequences for QS-related data.

## INTRODUCTION

Although *Vibrio cholerae*, the causative agent of cholera, has been studied for more than a century, we still lack important information needed to fully understand its environmental lifestyle, its transmission to humans, and its full pathogenic potential. In the context of pathogenesis, a plethora of studies have provided important information about its major virulence factors (e.g., the cholera toxin [Ctx] and the toxin-coregulated pilus [TCP]) (1–4). However, the identification of additional virulence factors to explain, for instance, the mild diarrhea caused by *ctx*-negative *V. cholerae* strains, as elucidated in an infant rabbit model of cholera, is still important (5). One such putative virulence factor is the recently discovered type VI secretion system (T6SS) of *V. cholerae* (6). The T6SS is a molecular spear used to transport toxic effectors to other Gram-negative bacteria or eukaryotes in a contact-dependent manner (7). The result of this intoxication is the killing of the adjacent cell if it does not exert immunity against the toxic effectors, as is the case for the attacker’s siblings (i.e., a kin-discrimination mechanism).

The T6SS has primarily been studied in two nonpandemic isolates of *V. cholerae* (strain V52, an isolate from Sudan, and strain 2740-80, a nontoxigenic isolate from Florida) that harbor a constitutively active T6SS. The cues leading to the production of this system in the pandemic O1 El Tor strains, however, remained largely unknown. In this context, we recently showed that the T6SS of several pandemic *V. cholerae* O1 El Tor isolates is induced upon growth on chitin (8), which is one of the primary niches of the pathogen in its natural aquatic habitat (9). In particular, we demonstrated that the T6SS is part of the chitin-induced natural competence regulon and is therefore coregulated with the DNA-uptake machinery of *V. cholerae* (8, 10).

**FIG 1:**
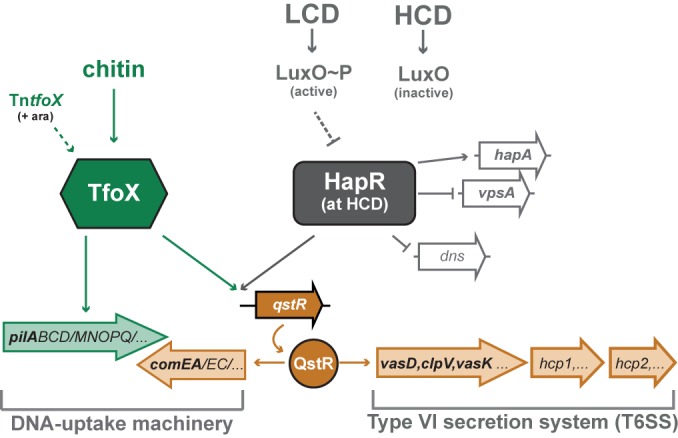
TfoX- and quorum-sensing (QS)-dependent regulation of the DNA-uptake machinery and the type VI secretion system (T6SS) in *V. cholerae*. The scheme shows the TfoX- and QS-dependent regulation of the competence regulon in *V. cholerae*, which includes genes encoding the DNA-uptake machinery and the T6SS. The activation of most genes requires dual input from chitin and a high cell density (HCD; compared with low cell density, LCD), which results in the production of the transformation regulator TfoX and the main regulator of QS, HapR, respectively. The signals of both of these proteins merge in the expression of *qstR*, which encodes the QS- and TfoX-dependent transcription factor QstR. TfoX, HapR, and QstR are required for the production of the essential parts of the DNA-uptake machinery and the T6SS (shown by orange arrows), whereas the type IV pilus part of the DNA-uptake machinery relies solely on the activation by TfoX (green arrow). QS-impaired C6706 mutant strains possess reduced HapR levels and, accordingly, reduced expression of the QstR-regulated genes. Natural transformation and T6SS-mediated interbacterial killing are therefore vastly impaired. The genes whose expression levels were measured in this study are in bold. LuxO~P, phosphorylated LuxO.

Natural competence for transformation is a widespread mode of horizontal gene transfer that is used by many prokaryotes to incorporate new genetic material into their own genomes (11, 12). Such genetic material is acquired through the uptake of external DNA via competence-induced DNA-uptake machineries (10). The sophisticated regulatory network that drives natural competence in *V. cholerae* has been studied for more than a decade (13), first by us and more recently also by others (reviewed in reference 14). Briefly, upon growth on chitin, the bacterium produces the main regulator of transformation, TfoX, which subsequently leads to the production of the type IV pilus part of the DNA-uptake machinery (15) (including the major pilin subunit PilA; Fig. 1). However, TfoX alone is not sufficient to allow DNA uptake to occur, as the induction of the second part of the DNA-uptake machinery (e.g., the protein ComEA, which pulls the DNA into the periplasm [16, 17], and the inner membrane transporter ComEC [15]) requires additional input from the quorum-sensing (QS) circuitry (18) (Fig. 1). This input occurs via the master regulator of QS, HapR, which itself is produced only at a high cell density (HCD) (for a review, see reference 19). Hence, HapR acts as a positive coactivator of chitin-induced natural competence. Additionally, HapR also represses the gene that encodes a nuclease (*dns*) (Fig. 1), which, if not repressed, has a major impact on natural transformation through the degradation of external and periplasmic DNA (16, 20, 21). Notably, the two input signals (e.g., HCD signaled through HapR and chitin signaled through TfoX) merge in the production of the QS- and TfoX-dependent regulator QstR (22), which is ultimately required for the production of the pilus-unrelated part of the DNA-uptake machinery (15–17) and for the induction of the T6SS (8) (Fig. 1).

Chitin-induced natural competence for transformation is conserved among *V. cholerae* strains as well as noncholera *Vibrio* species (such as *V. vulnificus* [23], *V. fischeri* [24], and *V. parahaemolyticus* [25]). However, despite this conservation and several reports that used our previously published protocol (26–28) or derivatives of it to genetically modify *Vibrio* strains, we frequently obtain requests from researchers who are unable to use chitin-induced natural transformation as a tool (especially for strain C6706; see below). The nontransformability of QS-defective strains, such as the first sequenced strain of *V. cholerae* N16961 (29), which contains an authentic frameshift mutation within *hapR*, was reported early on (13). The primary cause for the lack of natural transformation in this and other QS-defective strains is the absence of *dns* repression (Fig. 1), which results in constitutively high nuclease activity (20). Consistent with these data is a recent report that identified another transformation-inhibitory nuclease in a horizontally acquired integrative and conjugative element (ICE), which rendered such ICE-carrying strains similarly nontransformable (30). However, despite the fact that the well-studied *V. cholerae* C6706 strain (31), an O1 El Tor patient isolate from Peru, does not contain such an ICE and that this strain has been described as QS proficient and naturally transformable (13), several researchers have reported to us its nontransformability. Here, we followed up on this nontransformability by testing seven C6706 isolates obtained from different laboratories located in North America and Europe. We show that approximately half of these wild-type (WT) strains contain the same compromising mutation within *luxO*, resulting in impaired QS behavior and, consequently, in low natural transformability and T6SS activity.

## RESULTS

### The majority of C6706 strains are severely impaired in their natural transformability.

**FIG 2:**
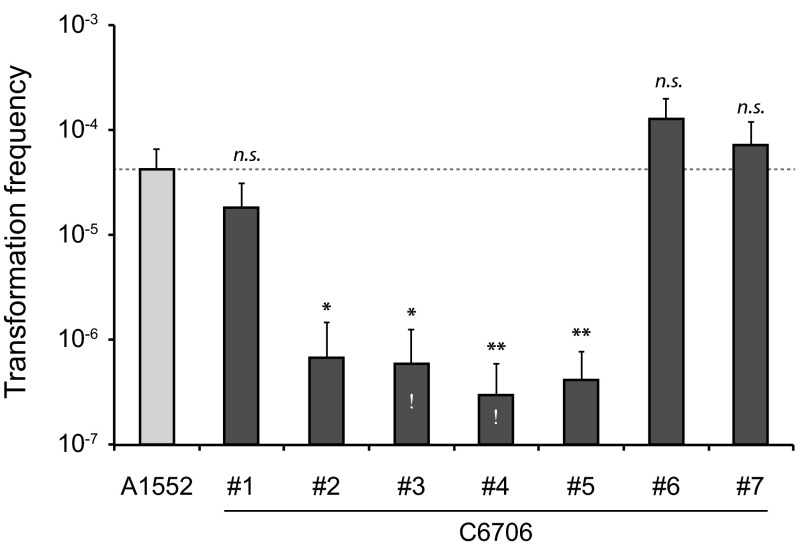
The seven samples of strain C6706 are transformation variable. The seven representatives of strain C6706 (and strain A1552 as a control) were grown on chitin flakes and scored for natural transformability. The data represent the average transformation frequencies of at least three biological replicates (±SD), and the dashed line shows the value for the A1552 control strain. If no transformants were recovered in a subset of the independent experiments, the detection limit value was used for calculations (indicated by the white exclamation mark). Statistically significant differences between the results from the different C6706 strains and the A1552 control strain were determined by Student’s *t* test (*, *P* < 0.05; **, *P* < 0.01; *n.s.*, not significant).

In 2005, it was shown for the first time that the human pathogen *V. cholerae* could enter a state of natural competence and that this phenotype depends on the presence of chitin (13). That study and follow-up studies showed that many patient isolates of *V. cholerae*, as well as environmental samples, are naturally transformable in a chitin-dependent manner (8, 13, 32, 33). However, frequent concerns exist in the field with respect to the transformability of O1 El Tor pandemic strain C6706 (personal communications from several researchers to M.B.). We therefore asked seven principal investigators working on diverse aspects of *V. cholerae* to share their C6706 strains with us. First, we tested these seven samples in a well-established chitin-dependent transformation assay (26) and compared the transformation frequencies to those of our main laboratory strain, the QS- and competence-proficient *V. cholerae* O1 El Tor A1552 strain. As presented in Fig. 2, our data confirmed that four of these seven wild-type C6706 strains were transformable only sparsely compared with the remaining three C6706 samples and the A1552 control strain. This bipartite response indicates that the nontransformability of such C6706 strains was not caused by an improper following of published protocols but rather by genetic differences between circulating C6706 strains.

### The majority of C6706 strains produce lowered *hapR* transcript levels and changed levels of expression of HapR-regulated genes.

**FIG 3:**
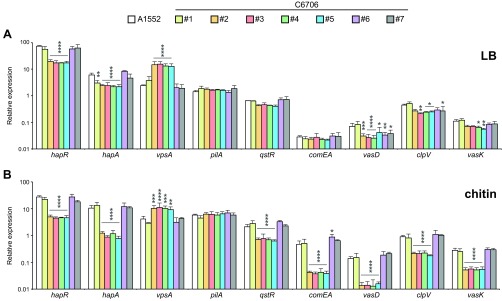
Two different patterns exist for the expression of QS-responsive genes in the seven C6706 samples. Data represent the relative expression levels of *hapR* and selected HapR-regulated genes (see Fig. 1 for details) as measured by qRT-PCR. Different *V. cholerae* C6706 strains (strain 1 to strain 7) were cultured in liquid LB medium to an HCD (A) or were statically grown on chitinous surfaces (B). Strain A1552 served as the positive control. The data represent the means (±SD) of the results of three independent biological experiments. Statistical differences between the A1552 control strain and the indicated C6706 strains (strain 1 to strain 7) were determined using two-way ANOVA. Only significantly different values are indicated: *, *P* < 0.05; **, *P* < 0.01; ***, *P* < 0.001; ****, *P* < 0.0001.

To elucidate whether the impairment of natural transformation was caused by a lack of chitin induction or by a problem in the QS circuit, we tested the seven C6706 strains for the expression of several QS-related and QS-unrelated genes, first in the absence of chitin (at HCD). In particular, we first monitored the transcript levels of *hapR* by quantitative reverse transcription-PCR (qRT-PCR) because the gene encodes an important coregulator of natural transformation and T6SS (Fig. 1). Interestingly, the *hapR* transcript levels were reduced in the same C6706 isolates (isolates 2 to 5) that also showed low transformability (Fig. 3A). These low *hapR* transcript levels were mirrored in lowered expression of *hapA* (for which HapR acts as an activator [34]) (Fig. 1) and in higher expression of the *Vibrio* polysaccharide synthesis gene *vpsA* (for which HapR acts as a repressor [35, 36]) (Fig. 1) than in the three highly transformable C6706 samples (sample 1, sample 6, and sample 7) and the A1552 control strain (Fig. 3A). The expression levels of competence-related genes did not differ between the samples under such chitin-independent HCD conditions (Fig. 3A), consistent with the fact that the competence regulon is not induced in LB medium in pandemic O1 El Tor strains.

When the same strains underwent a chitin-induced expression analysis, a similar QS-dependent pattern became obvious. As highlighted in Fig. 3B, the same differences in the *hapR* transcript levels were observed upon growth on chitinous surfaces, as described for LB-grown bacteria (Fig. 3A). The low levels of the HapR regulator were again reflected in the changed levels of expression of *hapA* and *vpsA* (Fig. 3B). Notably, while expression of the *pilA* QS-independent competence gene (Fig. 1) was induced in all of the tested strains upon growth on chitin (Fig. 3), the expression of the chitin- (TfoX-) and QS-coregulated competence genes was severely reduced in the same subset of QS-impaired C6706 strains, which were almost nontransformable (Fig. 2 and 3B). The affected genes were *qstR*, which itself requires induction by TfoX and HapR (22) (Fig. 1), and all of the tested QstR-dependent genes that encode either parts of the DNA-uptake machinery (e.g., *comEA*) or components of the T6SS (e.g., *vasD*, *clpV*, and *vasK*) (Fig. 3B). Notably, this striking difference in competence gene expression was observed even though we have previously demonstrated that chitin-attached bacteria show heterogeneity with respect to competence expression (18). We therefore conclude that the low HapR level produced in 4 of 7 of the C6706 isolates is not sufficient to properly induce the competence regulon, which includes the T6SS.

### QS-impaired wild-type C6706 strains show reduced TfoX-induced interbacterial killing.

**FIG 4:**
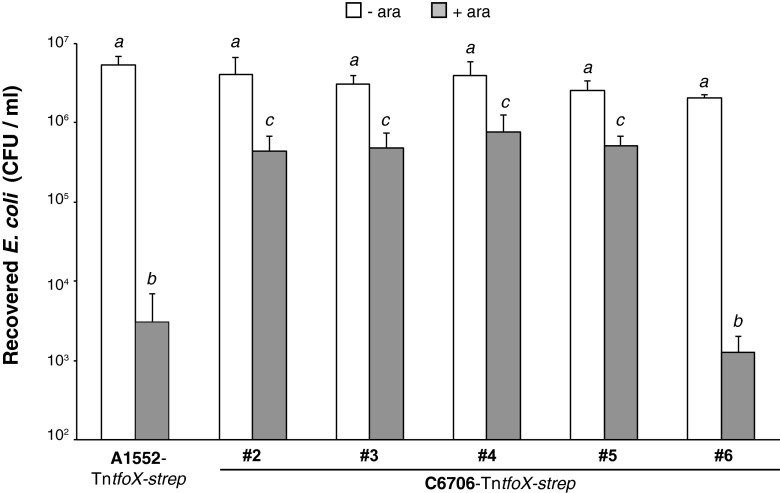
TfoX-induced interbacterial killing is vastly reduced in QS-impaired C6706 isolates. Data represent results of a killing assay of the indicated *V. cholerae* predator strains performed with *E. coli* as prey. Strain A1552 served as a positive control. All of the strains contained the Tn*tfoX-strep* transposon, which permits the induction of *tfoX* through the addition of arabinose (18, 37). The surviving prey were quantified as CFU per milliliter (*y* axis) after coculturing with the indicated *V. cholerae* strains on plain (-ara) or arabinose-containing (+ara) LB agar plates was performed. The averages of results from three biological replicates (±SD) are shown. Statistical differences were determined using two-way ANOVA. Data labeled with the same character (*a*, *b*, or *c*) are not significantly different. Differences between data labeled with different characters are significant (*P* < 0.0001 for comparisons between data labeled with *b* and data labeled with *c*).

Because we found decreased HapR levels in approximately half of the seven C6706 strains compared with the rest of the C6706 strains and the A1552 control, we considered whether this decrease was also reflected in a reduced ability to kill other Gram-negative bacteria, such as *Escherichia coli*, by means of T6SS-mediated attack. To answer this question, we introduced a transposon carrying an arabinose-inducible copy of *tfoX* (Tn*tfoX-strep* [18, 37]) into the chromosome of a representative set of C6706 strains, which allowed us to induce TfoX through the provision of the inducer (e.g., in a chitin-independent manner, as the latter condition does not support the growth of *E. coli*). We mixed these *V. cholerae* strains with an arabinose-non-degrading *E. coli* strain (37) and scored the recoverability of the *E. coli* prey after 4 h of coincubation. As shown in Fig. 4, QS-impaired C6706 strains 2 to 5 reduced the *E. coli* numbers only slightly, whereas the QS-proficient C6706 sample (sample 6) significantly reduced the prey population. This phenotype therefore reflects the expression analysis.

### QS-impaired wild-type C6706 strains possess a mutation in the QS gene *luxO*.

To elucidate the cause of the QS impairment and the low *hapR* transcript (and HapR protein) levels in a subset of the C6706 samples but not in the second half or in other O1 El Tor isolates, we sequenced two important QS genes: *hapR* itself and the gene encoding LuxO, the upstream-acting regulator of the *hapR* transcript (19). The rationale behind performing the latter was that at a low cell density (LCD), LuxO is in its phosphorylated and therefore active form. Phosphorylated LuxO (LuxO~P) indirectly (via the regulation of small RNAs) leads to the degradation of *hapR* mRNA (19) (Fig. 1). Importantly, and as shown in Table 1, all four of the QS-impaired C6706 strains contained the same G-to-A mutation in *luxO*, which resulted in an amino acid change from glycine to serine at position 333 of the protein (according to reference 38; annotated as glycine 319 initially [29]). None of the other three QS-proficient C6706 samples harbored this mutation, nor did any of the other O1 El Tor, O1 classical, or O37 strains (seven, two, and three isolates, respectively) which were tested at the same time (Table 1).

**TABLE 1:** Sequenced *luxO* and *hapR* genes in commonly studied *V. cholerae* strains

*V. cholerae* strain (serogroup)	Description of *luxO* gene^a^	Description of *hapR* gene^a^
A1552 (O1)	Wild-type sequence	Wild-type *hapR* sequence (without frameshift mutation, as is the case for N16961)
C6706#1 (O1)	Wild-type sequence	Wild-type sequence
C6706#2 (O1)	Mutant *luxO* (change of amino acid G333S^b^)	Wild-type sequence
C6706#3 (O1)	Mutant *luxO* (change of amino acid G333S^b^)	Wild-type sequence
C6706#4 (O1)	Mutant *luxO* (change of amino acid G333S^b^)	Wild-type sequence
C6706#5 (O1)	Mutant *luxO* (change of amino acid G333S^b^)	Wild-type sequence
C6706#6 (O1)	Wild-type sequence	Wild-type sequence
C6706#7 (O1)	Wild-type sequence	Wild-type sequence
N16961 (O1)	Wild-type sequence	Frameshift mutation in *hapR* confirmed (29)
N16961rep (O1)	Wild-type sequence	Wild-type sequence (frameshift mutation of strain N16961 repaired)
C6709 (O1)	Wild-type sequence	Wild-type sequence
E7946 (O1)	Wild-type sequence	Wild-type sequence
DRC-193A (O1)	Wild-type sequence	Wild-type sequence
P27459 (O1)	Wild-type sequence	Wild-type sequence
O395 (O1 classical)	Wild-type sequence	Frameshift mutation in *hapR* confirmed (39)
569B (O1 classical)	Wild-type sequence	Mutation in *hapR* (T169G leading to amino acid change Y57D)
V52 (O37)	Wild-type sequence	*hapR* mutation confirmed (premature stop codon) (43)
ATCC 25872 (O37)	Wild-type sequence	Wild-type sequence
ATCC 25873 (O37)	Wild-type sequence	Wild-type sequence

a Compared to the first sequenced strain of *V. cholerae*, N16961 (29).

b According to a changed annotation (38) (G333 corresponds to G319 in the original annotation provided for strain N16961 [29]).

## DISCUSSION

Here, we provide evidence for the circulation of a QS-impaired wild-type version of *V. cholerae* strain C6706. Four of seven tested C6706 samples obtained from international laboratories were severely affected with respect to their natural transformability and, accordingly, also with respect to their T6SS production and activity. Using qRT-PCR, we showed that the low transformability perfectly correlated with low levels of *hapR* transcripts and a changed expression pattern of several HapR-regulated genes. Accordingly, HapR-/QstR-coregulated competence genes (e.g., those encoding the DNA-uptake machinery and the T6SS) were not induced upon growth on chitin, explaining the compromised transformation and T6SS responses in these C6706 samples. Sequencing the *hapR* and *luxO* genes of the C6706 isolates and other O1 and non-O1 *V. cholerae* strains showed that the QS-impaired C6706 strains contained a mutation in *luxO*. Interestingly, the exact same mutation was recently described for a *luxU* mutant derivative of strain C6706 (38). In their study, Jung et al. provided evidence that the G333S amino acid change mimics the active form of LuxO (38). This change therefore explains why the mutated C6706 strains consistently had lower *hapR* transcript and protein levels than the nonmutated C6706 strains and non-C6706 control strain A1552.

An earlier study reported frequent mutations in the *hapR* gene of *V. cholerae* (39), and we speculated that such mutations are overrepresented in culture collections due to a sampling bias (40). Importantly, none of these O1 El Tor and classical isolates of *V. cholerae* contained the exact same mutation in *hapR* (39), excluding the clonal expansion of one successful mutant strain. Interestingly, however, Joelsson and colleagues mentioned in their study that the HapR protein level was reduced in strain C6706 compared with that in several other O1 serogroup strains (39), possibly caused by the here-described mutation of several wild-type C6706 strains. In this context, it should be noted that a recent study on *V. fischeri* showed that *luxO* mutations are frequently isolated from cultures in prolonged stationary phase (41). Importantly, the authors describe the isolation of a plethora of different *luxO* mutant alleles, all of which mimic the gene encoding a constitutively active LuxO protein (41). In our study, however, we found the exact same mutation in four different C6706 strains obtained from different laboratories, and this mutant allele of *luxO* exactly matches a previously reported mutation in a C6706-derived *luxU* mutant (38). Thus, it appears rather unlikely that the mutation arose independently in those five different strains. Instead, it can be assumed that the mutated C6706 strain was circulated among different laboratories. It is therefore of prime importance for any group studying QS-related phenotypes, such as the QS network, virulence expression, biofilm formation, natural competence for transformation, and T6SS production, in strain C6706 to ensure that the wild-type laboratory stock(s) (some laboratories seem to have more than one stock of the wild-type C6706 strain) and mutants thereof from other laboratories do not contain the previously reported (38) and here-described (for the WT) *luxO* mutation. Indeed, as we show in this study, for natural transformation and T6SS production/interbacterial killing, this mutation masks important QS-dependent phenotypes and therefore leads to the irreproducibility of such features.

It is unnecessary to mention that mutations can occur at any time. It is important that the presence of such mutations in commonly used strains is communicated within the scientific community in a timely manner to avoid unnecessary investments of time and resources using flawed reagents. Indeed, we have received numerous inquiries with respect to the nontransformability of C6706. The current study solved this mystery, as we revealed a QS-impairing mutation in a circulating C6706 strain. Our recommendation for researchers who work with *V. cholerae* C6706 is therefore to take measures to ensure that the *luxO* gene is not mutated in their laboratory stock.

## MATERIALS AND METHODS

### Bacterial strains and growth conditions.

The *V. cholerae* strains used in this study are listed in Table 2. *E. coli* strain TOP10-TnKan (37) served as prey in the interbacterial killing assay (see below). Bacteria were grown in liquid LB medium under shaking conditions or on LB agar plates (1.5% agar) unless otherwise stated. The temperature was kept at room temperature, 30°C, or 37°C. Half-concentrated defined artificial seawater (0.5× DASW [13]) was used for the chitin-induced natural transformation experiments. Antibiotics and other supplements were added at the following concentrations: kanamycin at 75 µg/ml and l-arabinose at 0.2%.

**TABLE 2:** Bacterial strains (*V. cholerae*) and plasmids used in this study

Strain or plasmid	Genotype/description^a^	Internal strain no.	Reference(s) or source
Strains			
A1552 (WT)	Wild type, O1 El Tor Inaba; Rif^r^	MB_1	44
A1552-Tn*tfoX-strep*	A1552 containing mini-Tn*7*-*araC*-P_BAD_-*tfoX-strep*; Rif^r^, Gent^r^	MB_3420	37
C6706 (strain #1–7)	*V. cholerae* O1 El Tor strain C6706; isolated in 1991, Peru; Str^r^ (31)	MB_1144 (#1), MB_1990 (#2), MB_2599 (#3), MB_3087 (#4), MB_3594 (#5), MB_3601 (#6), MB_4242 (#7)	Obtained from diverse laboratories in North America and Europe
C6706#2-Tn*tfoX-strep*	C6706#2 containing mini-Tn*7*-*araC*-P_BAD_-*tfoX-strep*; Str^r^, Gent^r^	MB_4146	This study
C6706#3-Tn*tfoX-strep*	C6706#3 containing mini-Tn*7*-*araC*-P_BAD_-*tfoX-strep*; Str^r^, Gent^r^	MB_4148	This study
C6706#4-Tn*tfoX-strep*	C6706#4 containing mini-Tn*7*-*araC*-P_BAD_-*tfoX-strep*; Str^r^, Gent^r^	MB_4150	This study
C6706#5-Tn*tfoX-strep*	C6706#5 containing mini-Tn*7*-*araC*-P_BAD_-*tfoX-strep*; Str^r^, Gent^r^	MB_4152	This study
C6706#6-Tn*tfoX-strep*	C6706#6 containing mini-Tn*7*-*araC*-P_BAD_-*tfoX-strep*; Str^r^, Gent^r^	MB_4154	This study
N16961	N16961, *hapR* frameshift; Str^r^	MB_2	29
N16961-rep	N16961, *hapR* frameshift repaired (TransFLP); Str^r^	MB_2254	45
C6709	*V. cholerae* O1 El Tor Inaba; isolated in 1991, Peru; Str^r^	MB_1503	46, 47
E7946	*V. cholerae* strain El Tor Ogawa; isolated in 1978, Bahrain; Str^r^	MB_2600	48, 49
ATCC 25872	*V. cholerae* non-O1 (O37); isolated in 1965, Czechoslovakia; Str^r^	MB_276	50, 51
ATCC 25873	*V. cholerae* non-O1 (O37); isolated in 1965, Czechoslovakia; Str^r^	MB_277	50, 51
DRC-193A	*V. cholerae* O1; patient isolate from 2011 (isolated at the Institut National de Recherche Biomédicale; Democratic Republic of the Congo); *ctxAB*^+^ *tcp*^+^ *hapR*^+^; Str^r^	MB_1954	8
P27459	*V. cholerae* O1 El Tor Inaba; isolated in 1976, Bangladesh; Str^r^	MB_1504	47, 52
V52	*V. cholerae* non-O1 (O37); Isolated in 1968, Sudan; Str^r^	MB_1510	47, 53
O395	*V. cholerae* O1 classical (Ogawa); Str^r^	MB_1147	54
569B	*V. cholerae* O1 classical (Inaba); Str^r^	MB_1148	51
Plasmids			
pUX-BF13	*ori*R6K, helper plasmid with Tn*7* transposition function; Amp^r^	MB_457	55
pGP704-mTn*tfoX-strep*	pGP704 with mini-Tn*7* carrying *araC* and P_BAD_-driven *tfoX-strep*; Amp^r^, Gent^r^	MB_3664	37

a Amp, ampicillin; Gent, gentamicin; Rif, rifampin; Str, streptomycin.

For the selection of *V. cholerae* after triparental mating performed with *E. coli* donor strains, thiosulfate citrate bile salts sucrose (TCBS) agar plates were used. The plates were prepared following the standard protocol provided by the manufacturer (Sigma-Aldrich/Fluka, Buchs, Switzerland).

### Natural transformation assays on chitin surfaces.

The natural transformability of the diverse *V. cholerae* strains was tested through an established transformation assay performed using chitin flakes (26, 27). The genomic DNA of strain A1552-lacZ-Kan (26) served as the transforming material. The frequencies were calculated as the number of kanamycin-resistant transformants divided by the total number of CFUs. The averages (±standard deviation [SD] as shown by the error bar) of results of four independent biological replicates are indicated in the figure. For calculation purposes, the value was set to the detection limit for the experiments that resulted in the absence of transformants (e.g., values below the detection limit of the assay). Significant differences between *V. cholerae* strain A1552 and the seven isolates of strain C6706 were evaluated with Student’s *t* test on log-transformed data (42).

### Gene expression analysis by quantitative reverse transcription-PCR (qRT-PCR).

For the LB growth conditions, the bacteria were grown at 30°C for 6 h under shaking conditions in liquid LB medium to reach a high cell density and processed as previously reported (8, 18). RNA that was extracted from chitin-grown bacteria was obtained by growing *V. cholerae* on chitin flakes (Sigma-Aldrich, Switzerland) (26). After 22 h of static incubation on chitin surfaces (for each strain in quadruplicate), the samples were centrifuged for 3 min, the supernatant was removed, and the pellet was resuspended in 1 ml of Tri Reagent (Sigma-Aldrich, Switzerland). After vortex mixing was performed to ensure homogenization, the samples were again centrifuged to remove residual chitin flakes. The supernatant was transferred to a new tube, shock-frozen on dry ice, and stored at −80°C.

The expression of representative genes was analyzed by quantitative reverse transcription-PCR (qRT-PCR) as previously described (18). The transcript levels of the indicated genes were normalized to the expression of *gyrA* to obtain the relative expression values. All of the experiments were performed three independent times, and averages (±SD) of results of all of the biological replicates are provided. Statistical analyses were based on two-way analysis of variance (ANOVA), which was performed using GraphPad Prism version 7 for Mac (GraphPad Software, San Diego, CA, USA).

### Interbacterial killing assay.

The interbacterial killing assay was performed as previously described (8) using *E. coli* strain TOP10-Kan (37) as the prey and the indicated *V. cholerae* strains as predators. The bacteria were grown in the absence (−ara) or presence (+ara) of 0.2% arabinose to induce the chromosomal copy on *tfoX* (harbored on transposon Tn*tfoX-strep* [18, 37]). Coincubation occurred at 37°C for 4 h with a ratio of predator to prey of 10:1. Recovered *E. coli* cells were enumerated through serial dilution followed by the counting of CFU per milliliter. Three independent experiments were performed, and averages (±SD) of results of these biological replicates are given in the figure. Significant differences were determined using two-way ANOVA (GraphPad Prism).
